# The Role of Brain Natriuretic Peptide as a Prognostic Marker for Sepsis

**DOI:** 10.7759/cureus.8954

**Published:** 2020-07-01

**Authors:** Binita Bhandari, Jessica Cunningham

**Affiliations:** 1 Internal Medicine, University of Pittsburgh Medical Center (UPMC) Pinnacle, Harrisburg, USA

**Keywords:** cardiac bnp, nt-probnp, sepsis, septic shock, prognostic marker

## Abstract

Brain natriuretic peptide (BNP) is a neurohormone released in response to volume expansion and increased pressure. It is commonly used to assist in the diagnosis and management of heart failure. BNP can also play an important role as a biomarker in septic shock; however, elevations of BNP in conditions other than sepsis or cardiac dysfunction limits its use as the sole prognostic marker in patients hospitalized with sepsis. Further relationships regarding laboratory value and correlation with severity of illness need to be established with larger prospective studies to develop consensus regarding a cut-off point for optimum sensitivity and specificity in predicting in-hospital mortality related to sepsis.

## Introduction and background

Natriuretic peptides are peptide hormones mostly released by the heart muscles in response to increased volume status and wall stress, which are key elements in cardiovascular physiology [1]. Brain natriuretic peptide (BNP) is a commonly measured natriuretic peptide, which is released in conditions that leads to volume expansion and increased myocardial wall pressure. The physiological functions of BNP include relaxation of vasomotor tone, inhibition of sympathetic activity, reduction in cardiac preload, increase in renal blood flow, and increase in natriuresis and diuresis [2].

Plasma BNP has become an important tool in clinical decision-making regarding diagnosis, management, and risk stratification of heart failure [3,4]. In addition to heart failure, it has also been studied in angina, pericarditis, and various non-cardiovascular conditions such as renal failure, hyperthyroidism, pulmonary embolism, anemia, and post-surgery [5-8]. Plasma BNP and N-terminal pro brain natriuretic peptide (NT-proBNP) are viewed as comparable in terms of the diagnosis, evaluation, and monitoring of heart-failure patients [9].

Sepsis is an inflammatory response to an infection characterized by systemic inflammation and life-threatening organ dysfunction [10]. It is a major cause of hospitalization and critical care admissions and is associated with a significant healthcare burden globally. In 2017, the new incidence of sepsis was estimated at around 48.7 million cases worldwide with 11 million sepsis-related deaths representing one-fifth of all global deaths [11]. Data from an international audit of the intensive care unit (ICU) patients have shown hospital mortality rates of up to one-third of patients with sepsis [12]. In the US, the cost of sepsis-related in-patient hospital admissions for Medicare beneficiaries has been on the rise, with an increase in total expenditure from $17.8 billion to $22.4 billion between 2012 and 2018. Combined with subsequent skilled nursing facility admission, the total Medicare cost is estimated to be $41.5 billion [13]. Due to the critical nature of sepsis, the associated morbidity and mortality, and the resulting economic impact on healthcare, prompt recognition of the condition and enactment of early therapeutic interventions in severe sepsis and septic shock are important.

## Review

Prospective studies have shown that plasma BNP concentration can reliably identify patients developing sepsis-induced myocardial depression [14]. There have been recent metanalyses looking at both BNP and NT-proBNP that have shown moderate predictive value for mortality in septic patients [15,16]. Larger prospective studies are required to establish further relationships regarding laboratory value and correlation with the severity of the illness to better identify a cut-off point for optimum sensitivity and specificity in predicting in-hospital mortality related to sepsis.

Patients with sepsis or septic shock have cardiovascular dysfunction. BNP is understood to be produced as a result of ventricular myocardial wall stretch, but other factors such as degree of inflammation, use of vasopressor medications as well as acute kidney injury may come into play in sepsis [17]. The primary mechanism for the elevation of BNP and NT-proBNP in critical sepsis remains uncertain.

Elevated BNP levels have been described even in the absence of demonstrable ventricular dysfunction and have been correlated with C- reactive protein levels [18], suggesting that the value may be an indicator of the severity of inflammation as well. Whether the mechanism for elevated BNP in critically ill patients with sepsis is solely due to cardiovascular insufficiency or is influenced by other factors, like systemic inflammation, remains unclear.

With regard to the correlation between plasma BNP levels and mortality, studies vary in terms of BNP cut-off levels and associated mortality. Charpentier et al. showed that BNP levels above 190 pg/ml in patients admitted with sepsis and septic shock were associated with a five-fold increase in the risk of death within 30 days [19]. Chen et al. used plasma BNP cut-off of 113 pg/ml in the emergency department, which independently predicted mortality in septic patients [20]. Perman et al. demonstrated a greater risk of mortality (11.6% vs 2.1%; p = 0.0001) and development of severe sepsis and septic shock with a lower cut-off level of 49 pg/ml [21].

Other studies have used higher cut-off values. For example, Papanikolaou et al. showed day-1 BNP of >800 pg/ml predicting mortality with area under the curve (AUC) value 0.7 (95% CI: 0.54-0.86) [22]. Similarly, Khoury et al. concluded that admission plasma BNP above 1,000 pg/ml independently predicted short- and long-term mortality in hospitalized patients with sepsis and septic shock [23]. The prognostic value of elevated BNP concentration regarding in-hospital mortality and length of hospital stay has been established regardless of the presence of cardiac dysfunction [24].

Conversely, some studies have failed to show a correlation between elevated BNP levels and mortality. Mclean et al. showed that BNP levels are elevated in sepsis and septic shock patients in the presence or absence of cardiac dysfunction; however, they did not show any prognostic significance of initial BNP levels or daily changes in BNP levels [25]. Cuthbertson et al. also showed elevated BNP levels in patients with sepsis and septic shock, but failed to show any predictive value in terms of mortality or outcome after intensive care [26].

Recent metanalyses looking at both BNP and NT-proBNP have shown moderate predictive value for mortality in septic patients [15,16]. Vallabhajosyula et al. analyzed 35 studies (3,508 patients) and concluded that a cut-off level of plasma BNP of 633 pg/ml shows the greatest discrimination for mortality with an area under the receiver operating characteristic (ROC) curve of 0.766 (95% CI: 0.734-0.797) [16]. However, as the authors have pointed out, the heterogeneity of the included patient population means that these results are not applicable to all the patients admitted with sepsis and septic shock.

## Conclusions

BNP can play an important role as a biomarker in septic shock; however, elevations of BNP in conditions other than sepsis or cardiac dysfunction limits its use as the sole prognostic marker in patients hospitalized with sepsis. BNP is relatively easy to obtain as a laboratory test and has the potential to identify patients with imminent cardiovascular compromise and those at high risk for mortality. Further relationships regarding laboratory value and correlation with severity of illness need to be established with larger prospective studies to develop consensus regarding a cut-off point for optimum sensitivity and specificity in predicting in-hospital mortality related to sepsis.
